# VA Connection Plans: A Whole Health Intervention to Promote Social Connections for Older Veterans

**DOI:** 10.1093/geroni/igab046.786

**Published:** 2021-12-17

**Authors:** Amanda Peeples, Samantha Hack, Anjana Muralidharan

**Affiliations:** 1 VISN 5 MIRECC, Baltimore, Maryland, United States; 2 VA Maryland Health Care System, Baltimore, Maryland, United States; 3 Veterans Affairs Capitol Healthcare Network, Baltimore, Maryland, United States

## Abstract

The Connection Plan intervention was created as a brief intervention to assist older adults experiencing social isolation during COVID-19. Based in Cognitive Behavioral Therapy (CBT), it is designed to help older adults create a “Connection Plan” to cope with distress related to social isolation. In 1-2 sessions, interventionists work with the older adult to create a Connection Plan with three parts: Mind (ways to change negative thoughts), Body (ways to change unpleasant body sensations), and Connections (ways to increase social engagement). Through soliciting feedback from key stakeholders (Veterans and VA clinicians), the Connection Plan intervention was adapted for the VA context. This paper will present this process of creating the VA Connection Plans manual, as well as associated efforts to disseminate the intervention to 900 VA staff and deliver it to 600 older Veterans with (age 50+) and without (age 65+) serious mental illness.

